# How is success achieved by individuals innovating for patient safety and quality in the NHS?

**DOI:** 10.1186/s12913-017-2589-1

**Published:** 2017-09-11

**Authors:** Laura Sheard, Cath Jackson, Rebecca Lawton

**Affiliations:** 1 Yorkshire Quality & Safety Research Group,Bradford Institute for Health Research, Bradford Teaching Hospitals, Duckworth Lane, Bradford, BD9 6RJ UK; 2 Valid Research Ltd, Sandown House, Sandbeck Way, Wetherby, West Yorkshire, LS22 7DN UK; 3 School of Psychology, University of Leeds, Leeds, LS2 9JZ UK

**Keywords:** Innovation, Positive deviance, Qualitative research, Patient safety, Healthcare organisations

## Abstract

**Background:**

Innovation in healthcare is said to be notoriously difficult to achieve and sustain yet simultaneously the health service is under intense pressure to innovate given the ever increasing demands placed upon it. Whilst many studies have looked at diffusion of innovation from an organisational perspective, few have sought to understand how individuals working in healthcare innovate successfully. We took a positive deviance approach to understand how innovations are achieved by individuals working in the NHS.

**Method:**

We conducted in depth interviews in 2015 with 15 individuals who had received a national award for being a successful UK innovator in healthcare. We invited only those people who were currently (or had recently) worked in the NHS and whose innovation focused on improving patient safety or quality. Thematic analysis was used.

**Findings:**

Four themes emerged from the data: personal determination, the ability to broker relationships and make connections, the ways in which innovators were able to navigate organisational culture to their advantage and their ability to use evidence to influence others. Determination, focus and persistence were important personal characteristics of innovators as were skills in being able to challenge the status quo. Innovators were able to connect sometimes disparate teams and people, being the broker between them in negotiating collaborative working. The culture of the organisation these participants resided in was important with some being able to use this (and the current patient safety agenda) to their advantage. Gathering robust data to demonstrate their innovation had a positive impact and was seen as essential to its progression.

**Conclusions:**

This paper reveals a number of factors which are important to the success of innovators in healthcare. We have uncovered that innovators have particular personal traits which encourage a propensity towards change and action. Yet, for fruitful innovation to take place, it is important for relational networks and organisational culture to be receptive to change.

## Background

Healthcare innovation has been defined as “a novel set of behaviours, routines and ways of working that are directed at improving healthcare outcomes, administrative efficiency, cost effectiveness or users’ experience and that are implemented by planned and co-ordinated actions” [1]. The healthcare sector in the United Kingdom is under continual pressure to innovate given the ever increasing efficiency demands placed upon it [2]. Yet, the challenges of implementation and diffusion mean that achieving sustained innovation in healthcare is notoriously difficult [3]. A number of interacting factors have been identified to underpin the speed of implementation and diffusion as well as its sustainability [4]. These relate to the innovation itself, the intended adopters, communication and influence, both the inner and outer organisational/system context and the process of implementation [1].

Ever since Roger’s seminal work on diffusion of innovations theory [4], a body of literature has emerged concerning how innovations are spread, diffused or implemented in many sectors including healthcare. Several recent papers have examined healthcare innovation in different ways. McMullen et al. (2015) used the diffusions of innovations model to understand the results of a trial which tested a novel rapid HIV test in UK primary care [5]. Illott et al. (2012) assessed the implementation of healthcare innovations arising from a region in the North of England [2]. Barnett et al. (2011) were interested in factors which obstructed or facilitated the implementation and diffusion of innovation in healthcare organisations [6]. Other research teams have looked at innovations in relation to a precise clinical area e.g. why transcatheter aortic valve implantation showed rapid diffusion in Germany but not elsewhere [7]. These studies have addressed the issue of diffusion and spread, but significantly less work has emphasised how innovation is developed and achieved or the role of individuals in producing healthcare innovations. Due to the paucity of knowledge in this area, our research team became increasingly interested in understanding how innovation is achieved by individuals in the NHS with regards to patient safety and quality.

Patient safety is a key government and NHS concern [8] but little is known about how individuals working within healthcare organisations are able to achieve innovations specific to patient safety and quality. Attempts to improve patient safety within healthcare organisations often rely on identifying when patient safety is compromised via methods such as mortality reviews, audits and incident reporting. The emphasis on ‘find and fix’ or “what goes wrong and how often, why errors occur, and who or what is at the root of the problem… tell us little about the presence of patient safety, alerting us instead to its absence” [9]. Despite this negativity, the majority of care that is delivered is of a high quality and safe [10]. Approaches which focus on strengths and resources - looking at why things go right in order to learn from success – are beginning to gain credence [9]. One such approach is that of ‘positive deviance’ [11]. Positive deviance has its roots in international public health research [12] but has recently begun to be applied to western healthcare settings to address patient safety topics such as reducing surgical site infections [13], and the promotion of hand hygiene [14]. A central tenent of the positive deviance approach is that solutions to problems facing a given community usually exist amongst certain members of that community, which can then be taken and spread to other members [12]. Despite facing similar constraints as others, ‘positive deviants’ are able to succeed by demonstrating different or uncommon behaviours [15]. Most studies which have identified and conducted work with positive deviants have been focused at the level of the organisation rather than the individual [15]. Of those studies which have focused on individual positive deviants in healthcare, most use quantitative methods [14, 16]. One exception is Kim et al. (2008) who identified the strategies used by positively deviant nurses (and patients) when focusing on communication about family planning programmes in Indonesia [17]. There is little other literature of a sufficient methodological quality regarding individual positive deviants. In this study, our aim is to understand how individuals working within the NHS manage to implement innovations which benefit patient safety. This is one of the first studies to use the positive deviance approach to examine how innovators for quality and safety in the NHS achieve success.

Our original research questions were:

-What do successful individuals believe helped them to achieve their innovation?
-What do innovators believe they do differently to others with a similar role and status?
-Do the innovators see themselves as unusual? How and in what ways?
-What approach to leadership do the innovators take?

## Methods

### Study design

A qualitative research design was employed, undertaking semi-structured in depth interviews with innovators in patient safety and quality. In depth interviews were selected because they often lead to rich narratives, which permit the researcher to analyse how the participants make sense of the topic under investigation [18]. As this study was exploratory in nature, we did not begin with an a priori hypothesis. We took a positive deviance approach whilst grounding ourselves in an applied health services research paradigm. Ethical approval for the study was granted by the University of Leeds in March 2015.

### Participants and recruitment

We set out to interview around 15 innovators working in the area of patient safety and quality, within the NHS. Potential participants were identified from the Health Services Journal (HSJ) supplement ‘Top 50 innovators in Healthcare’ in 2014 [19] and 2013 [20]. HSJ is a weekly journal read by healthcare staff and NHS managers. Potential participants were people who had won a national award for their work in innovation in healthcare. We selected participants to approach from the HSJ Innovators awards list whose innovation had made a substantial contribution to patient safety or quality (or had the potential to do so), as judged by all three authors. They also had to be a healthcare professional currently or recently working for the NHS. We excluded innovators if: their area was not patient safety or quality, their work had no direct impact on patients, their work related only to a technical invention, high level infomatics or cost effectiveness/cost reduction.

Twenty two potential participants were emailed by LS and invited to take part in an interview and 15 agreed to take part (68% response rate). For those individuals who responded, a participant information sheet and consent form were then e-mailed to them. An interview date was subsequently arranged. Participants completed and returned a consent form prior to the interview. As this study was with ‘elite’ participants, a small sample size is more appropriate than for a qualitative study undertaken with patients or the public [21]. We anticipated a priori that around 12–15 participants would be needed to draw general conclusions about innovators in safety and quality in healthcare [21]. Table 1 provides a short description of each participant’s innovation. The innovations described were varied. Although they all concentrated on patient safety and quality, some related to developing new processes or different ways of working whilst others sought to spread or scale up already existing ideas.

**Table 1:** The main innovation of participants

P1	Led the implementation and adoption of an organisational development approach within a NHS Hospital Trust, working with teams in difficulty.
P2	Took the concept of enhanced recovery in surgical patients and introduced it for medical patients.
P3	Improved the use of health informatics in the NHS, setting up systems to collect and review real-time performance data.
P4	Introduced the use of PROMs with patients undergoing orthopaedic surgery to improve the efficiency of follow-up clinics.
P5	Designed a new, safer technique for glaucoma surgery including anti-scarring treatment.
P6	Developed a rehabilitation pathway using digital media to engage patients more in the process.
P7	Redesigned patient pathways to improve outcomes for dialysis patients as well as deliver efficient and more sustainable dialysis services.
P8	Led the implementation and adoption of an organisation-wide intervention to improve patient safety in an NHS hospital trust.
P9	Scaled up and rolled out a tele-health initiative across a region of England
P10	Lead the implementation of specialist geriatric-led service for older elective surgical patients
P11	Developed a patient experience campaign focusing on improving communication between staff and patients
P12	Implemented and scaled up a novel method for stimulating successful communication in healthcare teams in order to improve patient safety outcomes
P13	Led a large scale reconfiguration of the centralisation of stroke services in a major city in the UK
P14	Developed an approach to improve the process of ward rounds in order to reduce errors relating to patient safety
P15	Led the transformation of primary care services in a major city in the UK

### Data collection

Fifteen interviews were conducted by LS and CJ between June and September 2015. LS is a medical sociologist and CJ is a health psychologist. Both have worked in health services research for almost fifteen years each and have significant qualitative expertise. Both are educated to doctorate level in their respective fields. Of the fifteen interviews, five interviews were conducted face-to-face in the offices of participants and ten were conducted over the telephone. The approach was dependent on the location and/or preferences of the participant.

All interviews were conducted using a topic guide to ensure consistency across participants; however, the format was flexible in order to allow participants to voice what they considered important. Interviews began with a discussion of the innovation which led to their HSJ award, then progressed to examining the levers around successful innovation, paying particular attention to the individual circumstances of the participant and their role (including leadership) in the NHS organisation where they worked. The behaviour change wheel [22] informed the interview questions. This considers capability, motivation and opportunity for behaviour (in this case, innovation). Using a positive deviance approach as outlined previously, we explored whether these innovators were doing anything differently to other clinicians, and if so, how they managed to become a positive deviant in terms of innovation. Interviews lasted between 20 and 50 min and were all digitally audio-recorded. The voice files were transcribed verbatim by a professional transcriber.

### Analysis

We employed thematic analysis [23]. This approach is both inductive (themes emerge from the data and are not imposed upon it by the researchers) and iterative (data collection and analysis occur simultaneously). The iterative nature of the analysis meant that preliminary insights gathered during fieldwork then assisted in partially shaping the resultant coding framework. Comparative analysis was also carried out; this method allows data from different participants to be compared and contrasted. Discordant cases were actively sought throughout the analysis and emerging ideas and themes were modified in response. Data analysis involved a process of organising the data, descriptive coding, interpretive coding, writing and theorising. Data were managed using a qualitative computer software package (NVivo) to aid sorting and categorisation of the data analysis process. LS and CJ developed the coding framework collaboratively after in depth readings of four transcripts considered typical of the dataset. CJ then coded all the transcripts and LS conducted the next level of analysis and writing. Agreement regarding interpretation was reached through consensus discussion.

## Findings

Four themes were identified from the analysis: a) personal determination b) brokering relationships and making connections c) navigating organisational culture d) using evidence to influence others. Below, we outline broad factors across the themes which allow for an exploration of what helped these participants achieve their innovations and potential ways in which they were deviating from the norm. The themes arose inductively from the interviews conducted with the 15 participants and each has been ascribed a number to protect their identity.

### Personal determination

In seeking to discover what allows for some individuals working clinically within the NHS to develop innovations on a national level, it can be said that these people are driven to an exceptional level to provide better care for their patients. They were persistent, energetic, determined and compelled to be a clinician that went consistently above and beyond their normal ‘day job’ in order to make real change within the health service. Most of the participants described their innovation as happening outside of their normal working hours and indeed some vocalised that it was largely impossible to innovate within the time constraints of an average clinical workload. Being single minded and focussed on what they wanted to achieve was a strategy described by many of the innovators. Taking on the role of an innovator was said to be hard and something that could easily be given up if the inner determination to succeed and perseverance was not fully present, with two participants remarking “you need grit to make change” (P15) and:

*It’s about believing in what you do and someone saying ‘you’re the guy with the bee in your bonnet’ (P4).*

Divergence existed in the sample as to whether these were a group of people who were constantly innovating (“serial innovators”) or whether they had one main idea which they sought to propel forward and spread. A few participants did not feel comfortable being referred to as innovators and somewhat rejected the label.

Three participants spontaneously described themselves as “mavericks”. Over half the sample talked about how they felt it was necessary to challenge the status quo during the course of developing or implementing their innovation – either directly or as a result of their innovation being unduly stymied by various factors. Challenging the status quo in order to bring about innovation was said to be difficult and had led to resistance from others. One participant remarked: “people don’t want the innovation, they want the status quo” (P1) whilst another said “people always think something new is wrong” (P5). Choosing to be known as an innovator was said to be hard and sometimes unwelcoming:

*I ended up being seen as a radical and a troublemaker rather than somebody who’s fighting every day to try and make it a little bit better for the patients and for the staff I work with (P14)*

It was felt that clinicians needed to be at a certain point of seniority in their career where “rocking the boat” (P3) or “going against the trend” (P7) was not perceived as too detrimental to their status amongst their colleagues and within their organisation. Overall, we observed distinct personal traits related to determination, focus, persistence and being able to challenge the status quo were essential to ensure innovation happened.

### Brokering relationships and making connections

Innovators often felt that a critical part of their role was as a broker in bringing teams together and, in a lot of cases, encouraging people in divergent specialties to talk to each other and work together. This was a critical part of the journey of how their innovation was achieved and this ‘broker’ role sometimes differentiated them from other clinical peers. The ability to connect different people and connect different ideas together was important. Part of this role was described by some as about being able to convincingly market their ideas to others with P3 stating “you have to be a politician and a salesman”. Sometimes this role was difficult if participants did not already have established networks and were having to track down the appropriate people to assist them. Several participants remarked that they had to step outside of their own clinical area in order to deliberately build up relationships with other specialities to move their innovation forward:

*[We’re] trying to show people how they can be innovative around services and how you can get collaboration going between specialities that haven’t traditionally collaborated, like [innovator’s team], anaesthetists and surgeons (P10).*

Innovations which required cross working from diverse sections of the health system were said to be difficult to implement or set up but fundamentally necessary.

Of importance to the spread of some innovations were the fostering of collaborations or relationships between different directorates or wards of the same hospital who may not usually work together. In some cases, this was through local clinical networks (both formal and informal) or by clinical peers informally attesting to the value of the innovation with each other. P12 described making her innovation as easy as possible for people to test on their ward in order to foster uptake. Many innovators spoke about social media and in particular the importance of Twitter in order to disseminate ideas around innovation. Some participants were already opinion leaders on Twitter and used this platform to engage others in their innovation idea whilst others took to Twitter and became recognised as a result of public attention garnered from their innovation. In the case of a few participants, Twitter activity propelled debate about their innovation onto a global platform.

Most participants discussed the wider team they resided in and how these people and their skills were integral to the success of the innovation. Comment such as “it could not have happened” without the wider team were prevalent throughout the sample. Many innovators directed praise and attention for the achievement of the innovation to the wider team in which they sat. For some, this involved feeling uncomfortable about being singled out to receive the innovation award as an individual when they felt that several others had contributed just as equally:

*I don’t like the attention that comes with it because I’d rather the attention be on the team and the data, like the fact that the team’s made a difference and the data is amazing, like in terms of the improvement. So I just feel uncomfortable with like individuals taking recognition for something that isn’t really about individuals (P12)*

### Navigating organisational culture

The setting and context in which participants worked was contentious in relation to whether they believed it had allowed them to succeed (or not) with regards to their innovation. In exploring this theme, it cannot be determined whether the ability to navigate organisational culture and use it to their advantage is part of how this group of participants deviate from the norm. Participants variously outlined how organisational politics (on a micro, meso and macro level) had impacted their attempts at innovation. Whilst the majority of the sample discussed this aspect of innovation, it was interesting to see that there was an equal split between whether participants believed the culture of the organisation had either facilitated or blocked their attempts to innovate. A minority of innovators discussed influential chief executives, medical directors or managers who had buoyed their ability to innovate. In a few of these cases, these participants were encouraged by superiors in order to intentionally provide provocation or disrupt normal proceedings. P2 gave the example of being selected for certain hospital committees in order:

*To challenge, hopefully not in an unpleasant way, but to raise the questions and bring ideas from elsewhere (P2).*

The context and circumstances in which innovators were navigating (or trying to navigate) organisational culture often played a large part in seeing their innovation to fruition. The safety and quality agenda was said to have come to the fore in the UK over the past ten years and five participants discussed how they felt this had facilitated their innovation. Some participants talked about the organisation in which they worked being culturally ready for change and the innovation taking hold because it was introduced “in the right place at the right time” (P13). This was particularly the case for innovations which involved large scale system change, which were sensitive to national policy or a change in governmental direction. It is interesting here to contrast the experiences of two participants - P13 and P14. P13 presided over a £20m radical structural reconfiguration of acute care which encountered comparatively little resistance despite anticipating the opposite. This contrasts with P14 who described feeling frustrated that his relatively small scale innovations were not taken on board by management within the organisation where he worked. This was despite some of these innovations – mostly relating to improving processes on hospital wards – achieving international attention and being implemented at hospitals in several other countries.

Related to P14’s experience, several participants iterated how hierarchy and bureaucracy in the NHS has impeded their innovative spirit and, in a few cases, their ability to spread the innovation they had created. Amongst this group of six participants, there was a sense of frustration about how, at times, it seemed exceptionally difficult to be able to embed or spread new ideas or practices. This was sometimes related to the nature of the innovation itself if it sought to change culture within healthcare. Responsiveness to innovation within the NHS was said to be slow and clunky, with many regulatory hurdles to overcome, as P15 describes:

*The difficult thing is the timescale that the bureaucracy works at is about a thousandth of the time that I work at. So it’s very frustrating to know what needs to be done and to have to wait a year whilst all the dots are dotted and the crosses are crossed. I think this is the story of the NHS really…I think innovation now, the system is such a mess, it’s so muddled and sticky (P15)*

Coupled with the above was the issue of stability and sustainability within innovative spaces. There was said to be a lack of a culture to nurture wider sustainability for innovation. Sometimes the emphasis was put on the individual who had made the innovation to sustain it long term which was seen as unfeasible by innovators themselves, as P7 remarks:

*Just because I can put in a lot of extra time cos I believe in it, that can’t be the model of sustainability. Somebody has to take it off you and create sustainability around it; in the NHS nobody does that. So when you’ve done something and it seemed to have worked, they’ll expect you to carry the can (P7)*

A few participants remarked that it was dangerous for just one person or a small group of people to be pushing an innovation forward as the sustainability of the innovation was liable to collapse if it was not embedded more widely. Finances were often integral to the above issue and a couple of participants remarked how there was a lack of willingness to take innovation forward by the organisation if little or no money was attached to it. Conversely, one participant had achieved re-design of a hospital service by using small pots of money and building up incrementally in order to show that the new model worked before applying for large scale funding.

An additional factor which stymied innovation was resistance from clinical peers who did not see the benefit, think it would make any difference or that it would be possible to introduce. This approach had frustrated some participants when the ultimate benefit of testing out the innovation was to improve patient safety or care. Some peers were said to not be willing to step outside their comfort zones in order to try new ways or processes of working. Particular reticence was said to be encountered when innovations sought to change clinicians’ ways of thinking. P10 outlines the resistance she experienced at first:

*When we first started there was a lot of resistance; I don’t think, you know, people really understood why or how [team] could add value to something as high tech as surgery and anaesthesia…would we be taking a lead where we shouldn’t be taking a lead? How that was going to infringe on their own clinical areas…Some of the consultants initially said that they didn’t want us seeing their patients (P10)*

Notable exceptions existed to the above with P12 being surprised at the ease with which her innovation was adopted by clinical peers throughout the hospital and then the wider region. P11 seemed amazed at the speed and coverage her innovation took hold in the public consciousness and throughout healthcare nationally and internationally. It is important to note that few participants spoke about how their innovation had been completely curtailed by bureaucracy or a lack of sustainability, although there was a sense that some innovations progressed slower than would have been desired.

### Using evidence to influence others

Half the sample discussed the role of data (usually statistical or quantitative) to support the robustness of their innovation. The predominant use of data was often cited as “evidence” to demonstrate to peers and others that the innovation had a positive impact on patient safety or quality and therefore should be taken forward by others. Five participants attested to increasing attention being given to their innovation when peers saw the difference it was making to clinical care or processes via the presentation or publication of hard data. This was often seen as incremental attention over time as data started to show that the innovation was successful. P12 demonstrates several of the above points:

*Quite quickly we started to see results, improvement in reducing falls, and that gave a massive sort of momentum and belief that it makes a difference. And then basically we then got lots of natural spread, probably six months later...I was confident we’d got data to say to my colleagues, can you do it seven days a week, you know, five days a week we’ve achieved this, imagine what we could do if we did seven days a week?... Normally you send an email asking people to do something, and you’ll get like a flurry of resistance. But there was no resistance and a few people said, you know “Great data, of course I’ll do that.” (P12)*

Some participants were able to demonstrate statistically significant statistics such as reductions in mortality, reduced hospital admissions, shortened lengths of stay and significant improvement in falls data. These results had been persuasive in the spread of the innovation regionally and nationally. Getting quick, robust data which demonstrated the innovation worked was influential to some participants whereas others worked for several years to build up a robust dataset before they could show the innovation had positive impact. Several participants mentioned the importance of ensuring that data collection processes were built into the testing or roll out of the innovation.

## Discussion

In this study, we used the positive deviance approach to learn from individuals who are high profile innovators within the NHS in order to explore their perceptions of how they achieved their innovation. When investigating ‘what works’ at the level of individual, we found that the main factors were around: personal determination, the ability to connect people and teams, the ways in which innovators were able to use organisational culture to their advantage and their ability to use evidence to influence others. It is important to acknowledge that determination, focus, persistence were important personal characteristics as were skills in challenging the status quo. Innovators were able to connect sometimes disparate teams and people, being the broker between them in negotiating collaborative working. Some participants were able to use the culture of their organisation and the current patient safety agenda to their advantage (others found organisational culture stifling and this is discussed further below). Gathering robust data to demonstrate that their innovation had a positive impact was seen as essential to its progression.

The majority of these themes are reflected in the broader literature on how change takes place in organisations. For example, the Consolidated Framework for Implementation Research [24], − which is a meta-theoretical conceptual framework that synthesizes constructs from theories about innovation, organisational change and knowledge translation, amongst others - is particularly relevant. Two of its five core constructs are pertinent: inner setting and characteristics of individuals. The inner setting construct outlines how important the quality of social networks and informal communication are within an organisation. Relationships between individuals and a sense of ‘community’ are said to sometimes be more important than individual attributes. This relates heavily to our finding that the ability of our participants to connect people and teams was often intrinsic to the success of their innovation. However, the authors of the framework [24] state that personal attributes such as motivation, values, capacity and intellectual ability have received inadequate attention by those interested in how change is implemented. A major theme arising from this study was about the personal attributes and, essentially, the personal determination which this group of participants had to push forward their innovation. The fact that these intrinsic traits have been little studied in relation to organisational change is perhaps not surprising given that it may seem ‘common sense’ that what allows individuals to achieve innovation is their inherent sense of self. However, this study has shown which personal traits the participants identified themselves as being important for innovative practice.

It is interesting to see that although our explicit focus was on the positive, many of the participants spontaneously discussed what had hindered their particular innovation or, more generally, their innovative creativity or practice. This negative element of the findings can be derived across the main themes although mainly relates to that of ‘organisational culture’ particularly issues of bureaucracy, sustainability and resistance from peers. We felt it was important for the content of the themes to arise inductively from the dataset and so we have paid attention to describing the holistic account of what participants felt was important to them when asked to talk about how they achieved their innovation. But in doing so we have unveiled elements of ‘the way things are done’ in the NHS which frustrated – and sometimes hindered – a number of our participants. Of interest is the fact that these participants were working at a high level and usually mostly in influential positions. Therefore, the implications for how innovations take hold and spread within the health service needs thought and attention paid to it if those at the top of the structure have difficulty. This ties into the wider literature about the inability of shop floor NHS staff to make improvements to services [25]. Sometimes, it not enough for a clinician to simply have a great idea and the determination to succeed. Success is often related to the complex interplay of services from different disciplines working together and a culture of ‘organisational readiness’ to change [26].

Our overt focus was on innovators who were working in the patient safety and quality field. It is therefore interesting that despite being encouraged to talk during the in depth interviews about the specifics of their innovation, many of the main messages arising from the findings section are general in nature and could apply to innovation in disparate fields. Two of the themes are of particular interest here – brokering relationship and making connections; navigating organisational culture. For instance, innovators were often trying to influence groups of wider colleagues with whom they may not ordinarily interact to address specific clinical issues. Therefore, the importance of relational aspects of the innovation was voiced as integral to its perceived success by our group of participants. That the findings are general rather than specific is useful as this may allow for these insights to be applicable to those working in healthcare areas other than patient safety and quality.

The concept of leadership was part of our original focus and one of the a priori research questions. The topic guide contained several questions about leadership and participants answered these questions to varying degrees. However, when analysing the dataset we did not get the impression that leadership per se was a point of interest to these participants. In fact, leadership was rarely spontaneously mentioned and answers to the topic guide questions about leadership were sometimes perfunctory. Therefore, we did not include leadership as a theme within our write up. Contemporary literature on leadership within the NHS highlights a discursive move from previous forms of traditional hierarchical leadership to new forms of distributed leadership where people have the skills and relationships to work across multiple levels and with a variety of stakeholders [27]. Martin & Learmonth (2012) [28] note that leadership is said to be no longer exercised by just those in formal positions of authority but “something to be brought out across and beyond the health service”. For major change to be embedded in a healthcare system, it has been shown that elements of both hierarchical and distributed leadership are necessary [29]. It is interesting that none of our participants explicitly spoke about distributed leadership as an approach they take. Yet, in describing how they approached the task of delivering the innovation, this is the style of leadership most participants implicitly adopted in order to connect people and teams, sometimes in challenging situations or environments.

The findings of this paper suggest that innovations are not easy to achieve in the current NHS and that innovation is certainly not conceived as part of the ‘day job’ of clinicians. When seeking those people for roles that require innovation, recruiters may benefit from paying particular attention to the ability of applicants to connect people and to challenge the status quo. Determination and a sense of moral responsibility to improve patient care may also be traits worth examining. Lessons for NHS organisations also emerge from these data. At a senior level, recognition of and support for innovators to bring different people together to address a problem may be key, particularly where this can bypass bureaucracy that might otherwise stymie progress**.** Indeed this is one of seven recommendations for diffusing innovations outlined by Berwick (2003) [30]. The problem remains, however, how can those working in healthcare be supported to become innovators? One approach is to provide an opportunity for aspiring innovators to be embedded within a community of similarly minded peers at a local and national level. This approach is reflected in the Q community [31] established by NHS England and the Health Foundation to improve the quality of health and care services. The ability to call on similar others for advice and the opportunities for learning together that such a community might provide could help future innovators to overcome the challenges they so often face. This study has important implications, recognising, as it does, the importance of relationships across the healthcare setting for making change happen. As well as supporting innovators, leaders in healthcare organisations could engage in strategies to encourage a culture of innovation. These might include opportunities for multi-disciplinary training and projects and engaging frontline staff in improvement projects. Other initiatives such as shadowing across staff groups, improvement training and mentoring that encourage a better understanding of the health system as a whole, may well serve to help innovations develop and flourish.

### Strengths and limitations

To our knowledge, this is the first study to take a positive deviance approach to understand how innovations which benefit patient safety are achieved within the NHS. We interviewed a sample of exceptional innovators who were able to provide insight at the highest level of innovation in patient safety – across a range of types of innovations in different clinical areas. One limitation of our study could be the small sample size although, as previously described, interviewing ‘elite’ participants allows for this. In taking a positive deviance approach, we were explicitly looking for the factors which allow innovations to succeed in the NHS. It could be that had we conducted a more traditional ‘barriers and levers’ style study (such as Barnett et al. [6]) which paid equal attention to what discourages or prevents innovation then we would have gained a more rounded view of patient safety and quality innovation. However, whilst describing the process of innovating, participants did articulate the hurdles they had to overcome. Perhaps by describing how they achieved this we learn more about what is required by innovators, their colleagues and managers to deliver improvements to quality and safety. Finally, our sample of participants were those drawn from a national award list of NHS innovators, representing highly successful and – for the most part – influential people. We may have garnered different answers to our research questions if we had interviewed participants who were perhaps not known on a national level and were delivering innovations with a smaller impact or reach.

## Conclusion

We interviewed patient safety and quality innovators using a positive deviance approach to understand how they achieve success. The main factors identified were: i) personal determination of the individuals including their ability to challenge the status quo, ii) their capacity to connect people and teams, and encourage collaborative working, iii) the ways in some which innovators were able to use organisational culture to their advantage and iv) using evidence to influence others. Whilst innovation in healthcare is said to be difficult to achieve, we have uncovered a number of the key aspects which we believe may lead to successful innovation by individuals working in the NHS. These findings can be used by those who recruit, train and support potential innovators in the NHS and those who are responsible for setting the policy agenda. For successful innovation to occur, both the relational and structural position of the innovator are critical.
